# Astrocyte Ezrin defines resilience to stress-induced depressive behaviours in mice

**DOI:** 10.1093/nsr/nwaf480

**Published:** 2025-11-04

**Authors:** Si-Si Lin, Bin Zhou, Si-Le Liu, Xing-Ying Ren, Jing Guo, Jing-Lin Tong, Bin-Jie Chen, Ruo-Tian Jiang, Alexey Semyanov, Chenju Yi, Jianqin Niu, Peter Illes, Baoman Li, Yong Tang, Alexei Verkhratsky

**Affiliations:** International Joint Research Centre on Purinergic Signalling of Sichuan Province /Research Centre on TCM-Rehabilitation and Neural Circuit, School of Acupuncture and Tuina/Health and Rehabilitation, Chengdu University of Traditional Chinese Medicine, Chengdu 611137, China; Laboratory of Anaesthesia and Critical Care Medicine, Department of Anaesthesiology, Translational Neuroscience Centre, West China Hospital, Sichuan University, Chengdu 610041, China; International Joint Research Centre on Purinergic Signalling of Sichuan Province /Research Centre on TCM-Rehabilitation and Neural Circuit, School of Acupuncture and Tuina/Health and Rehabilitation, Chengdu University of Traditional Chinese Medicine, Chengdu 611137, China; International Joint Research Centre on Purinergic Signalling of Sichuan Province /Research Centre on TCM-Rehabilitation and Neural Circuit, School of Acupuncture and Tuina/Health and Rehabilitation, Chengdu University of Traditional Chinese Medicine, Chengdu 611137, China; International Joint Research Centre on Purinergic Signalling of Sichuan Province /Research Centre on TCM-Rehabilitation and Neural Circuit, School of Acupuncture and Tuina/Health and Rehabilitation, Chengdu University of Traditional Chinese Medicine, Chengdu 611137, China; International Joint Research Centre on Purinergic Signalling of Sichuan Province /Research Centre on TCM-Rehabilitation and Neural Circuit, School of Acupuncture and Tuina/Health and Rehabilitation, Chengdu University of Traditional Chinese Medicine, Chengdu 611137, China; Department of Forensic Analytical Toxicology, School of Forensic Medicine, China Medical University, Shenyang 110122, China; Laboratory of Anaesthesia and Critical Care Medicine, Department of Anaesthesiology, Translational Neuroscience Centre, West China Hospital, Sichuan University, Chengdu 610041, China; College of Medicine, Jiaxing University, Jiaxing 314001, China; Research Centre, Seventh Affiliated Hospital of Sun Yat-sen University, Shenzhen 518107, China; Department of Histology and Embryology, Chongqing Key Laboratory of Neurobiology, Third Military Medical University, Chongqing 400038, China; International Joint Research Centre on Purinergic Signalling of Sichuan Province /Research Centre on TCM-Rehabilitation and Neural Circuit, School of Acupuncture and Tuina/Health and Rehabilitation, Chengdu University of Traditional Chinese Medicine, Chengdu 611137, China; Acupuncture and Chronobiology Key Laboratory of Sichuan Province, Chengdu 611137, China; Rudolf Boehm Institute of Pharmacology and Toxicology, University of Leipzig, Leipzig 04109, Germany; Department of Forensic Analytical Toxicology, School of Forensic Medicine, China Medical University, Shenyang 110122, China; International Joint Research Centre on Purinergic Signalling of Sichuan Province /Research Centre on TCM-Rehabilitation and Neural Circuit, School of Acupuncture and Tuina/Health and Rehabilitation, Chengdu University of Traditional Chinese Medicine, Chengdu 611137, China; Acupuncture and Chronobiology Key Laboratory of Sichuan Province, Chengdu 611137, China; International Joint Research Centre on Purinergic Signalling of Sichuan Province /Research Centre on TCM-Rehabilitation and Neural Circuit, School of Acupuncture and Tuina/Health and Rehabilitation, Chengdu University of Traditional Chinese Medicine, Chengdu 611137, China; Department of Forensic Analytical Toxicology, School of Forensic Medicine, China Medical University, Shenyang 110122, China; Research Centre, Seventh Affiliated Hospital of Sun Yat-sen University, Shenzhen 518107, China; Acupuncture and Chronobiology Key Laboratory of Sichuan Province, Chengdu 611137, China; Faculty of Biology, Medicine and Health, The University of Manchester, Manchester M13 9PL, UK

**Keywords:** Ezrin, major depressive disorder, astrocyte, astrocyte atrophy, astroglial cradle, resistance

## Abstract

Astrocyte atrophy is the main histopathological hallmark of major depressive disorder (MDD) in humans and in animal models of depression. Here we demonstrated that manipulating Ezrin expression specifically in astrocytes significantly increases the resilience of mice to chronic unpredictable mild stress (CUMS). Overexpression of Ezrin in astrocytes from the medial prefrontal cortex (mPFC) rescued depressive-like behaviours induced by CUMS, whereas down-regulation of Ezrin in astrocytes from the mPFC increased mouse susceptibility to CUMS and promoted depressive-like behaviours. These behavioural changes correlated with astrocytic morphology. Astrocytes from the mPFC of mice sensitive to CUMS demonstrated significant atrophy; similar atrophy was found in astrocytes from animals with down-regulated Ezrin expression. On the contrary, morphology of astrocytes remained unchanged in animals resistant to CUMS and in animals with astrocytic overexpression of Ezrin. Morphological changes also correlated with Ezrin immunoreactivity, which was low in mice with depressive-like behaviours and high in mice resistant to stress. We conclude that Ezrin-dependent morphological remodelling of astrocytes defines the sensitivity of mice to stress; high Ezrin expression renders them stress resilient, whereas low Ezrin expression promotes depressive-like behaviour in response to chronic stress.

## INTRODUCTION

Psychiatric diseases in general, and diseases of mood in particular, arise from an aberrant functionality of neuronal networks associated with pathological changes in neuronal excitability, synaptic transmission, synaptic plasticity, network architecture, and nervous tissue homeostasis [1–5]. Uncompensated chronic stress is one of the key aetiological factors in major depressive disorder (MDD) and stress-induced depressive behaviours; notably, stress affects structures in the medial prefrontal cortex (mPFC), which is instrumental in stress processing [6]. These structural changes reflect loss of tissue homeostasis and are manifested by synaptic loss and decrease in neural cell densities [7]. Emergence of depressive symptoms and behaviours are, however, highly individual and the same amount of stress produces different outcomes in both patients and animal models [8–10]. Resilience is an adaptive response reflecting the ability of the brain to tolerate environmental stress without pathological changes [11,12].

Environmental challenges and stress instigate adaptive/maladaptive remodelling of the nervous tissue, which heavily relies on neuroglia, the main homeostatic and defensive arm of the nervous system [13]. Depression in patients, as well as depression-like behaviours in animal models of major depressive disorder and post-traumatic stress disorder (PTSD), are characterized by a prominent decrease in the density and size of astrocytes, which arguably limits the allostatic capacity of the nervous tissue, impairs synaptic transmission, and facilitates the

development of aberrant mood [14–17]. Manipulations with astrocytes and their homeostatic pathways (astrocytes ablation, down-regulation of astroglia-specific glutamate transporters or gap junctions) are sufficient to trigger depressive-like behaviours in rodents, whereas manipulating neurones does not have such an effect [17–20]. Moreover, stimulating glial glutamate uptake with riluzole or ceftriaxone alleviates depressive-like behaviours and increases astrocytic density [21,22]. Similarly, treatment with anti-depressive drugs or with acupuncture rescues depressive behaviours and restores astrocytic morphology [23]. These findings indicate that mood disorders, including stress-induced depression and MDD, are primary astrocytopathies linked to astrocytic atrophy and loss of function.

Astrocytes (which belong to a broader class of astroglia) are principal homeostatic cells of the central nervous system (CNS) supporting nervous tissue at all levels of organization, from molecular to organ-wide [24,25]. Protoplasmic astrocytes populating grey matter are characterized by a complex spongiform morphology with several principal processes or branches emanating from the soma; these processes give rise to branches of higher degree and terminal arborization made from tiny (∼100 nm in thickness) leaflets [26,27]. Morphological plasticity of these leaflets is regulated by the plasmalemmal–cytoskeleton linker Ezrin, involved in filopodia formation and astrocyte process motility [28–30]. Astrocytic leaflets and, less often, primary branches associate with synapses and form the synaptic cradle that fosters and sustains synaptic transmission [31]. Astrocytes regulate synaptogenesis, support synaptic transmission through numerous transporters that control neurotransmitters, provide ionostasis, supply neurotransmitter precursors and energy substrates, and regulate synaptic extinction [13,31–35]. Thus astrocytic atrophy limits homeostatic support and hence impairs the synaptic transmission.

Astroglial contribution to neuropathology is complex and mutable; pathophysiology of astrocytes ranges from reactive astrogliosis to astrocytopathies, astrocytic atrophy with loss of function, astrocytic degeneration and astrocytic death [36]. Astrocytic atrophy is manifest in ageing [37,38], neuropsychiatric disorders [39,40], neurodegenerative disorders [41], epilepsy [42,43], aberrant social behaviour [44], fear memories [45] and addiction [46]; the loss of astrocyte homeostatic support is one of the leading mechanisms of neuronal damage and death across many neurological diseases [36]. Retraction of astrocytic leaflets is of particular importance, as it affects synaptic support and causes aberrant synaptic transmission and neuronal excitability. Chronic unpredictable mild stress (CUMS) is generally employed to generate rodent models of depression [47,48]. Exposure to various stress regimens, including CUMS, does not trigger depressive-like behaviour in all mice; a sub-population (15%–50%) of animals demonstrate resilience to stress, which arguably reflects various adaptations [10,49–52]. Morphological examination of CUMS-induced depressed mice revealed significant atrophy of astrocytes, manifested by a decrease in glial fibrillary acidic protein (GFAP)-positive profiles, as well as astrocytic domains visualized with specific expression of genetic fluorescent probes [23,53,54].

In our previous study we found a correlation between astrocytic atrophy, down-regulation of Ezrin, and depressive-like behaviours [23], suggesting a causal link between Ezrin and the pathophysiology of depression. In the present study, we manipulated astrocytic Ezrin expression and analysed astrocytic morphology using high-resolution morphological reconstructions and behaviour of mice subjected to a CUMS regimen. We found that Ezrin overexpression not only prevented astrocytic atrophy but also significantly increased mouse resilience to stress. Our work suggests the key role of Ezrin and astrocytic leaflets in the pathophysiology of depressive disorders and defines Ezrin as a target for managing stress-related depression.

## RESULTS

### Astrocytic Ezrin expression defines resilience to chronic stress

**Figure 1.**
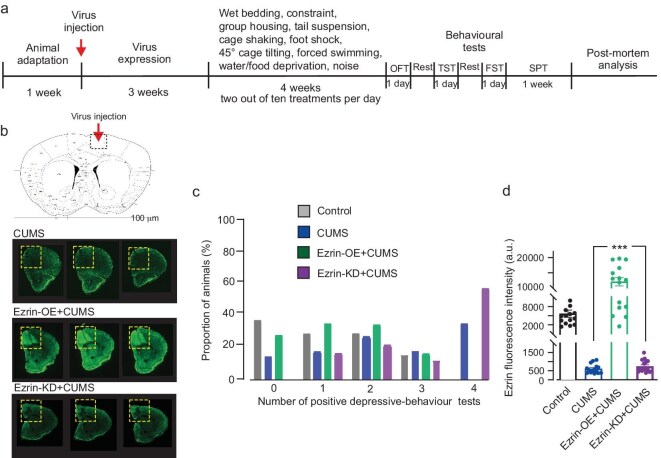
Astrocytic Ezrin expression defines mice resilience/sensitivity to susceptibility to chronic stress. (a) Experimental protocol. (b) The map of mouse brain with the virus injection site in the right mPFC and immunostaining of the mPFC with anti-Ezrin antibodies showing efficacy of both overexpression and knock-down of Ezrin in astrocytes. (c) Proportion of animals (in % of the respective groups) demonstrating 1, 2, 3 or 4 positive depressive-like behaviour tests following 4 weeks of the chronic stress regimen. (d) Expression of Ezrin was quantified by immunocytochemistry in mPFC slices in untreated controls, CUMS, Ezrin-OE + CUMS and Ezrin-KD + CUMS animal groups. ***P < 0.001.

To evaluate the role of astrocytic Ezrin in mouse response to stress we subjected three experimental groups to a CUMS protocol: (i) mice that received an injection of a vector with mCherry to label astrocytes (CUMS group; *n* = 103); (ii) mice with astrocyte-specific overexpression of Ezrin (Ezrin-OE + CUMS; *n* = 20); and (iii) mice with astrocyte-specific knockdown of Ezrin (Ezrin-KD + CUMS, *n* = 20). Both overexpression and knockdown were induced by relevant viral-carried constructs, as described in the Materials and methods section (experimental design is shown in Fig. 1a). Virus was stereotaxically injected into the right mPFC, as shown in Fig. 1b; the efficacy of Ezrin overexpression and knockdown as revealed by immunocytochemistry are shown in Fig. 1b and Fig. S1. We also set up the control group, which received an injection of a vector with mCherry to label astrocytes but were not exposed to CUMS (control group, *n* = 23). After 4 weeks of mouse exposure to CUMS behavioural tests and post-mortem analyses were performed (full account of behavioural tests is summarized in Table S1). The tests included: (i) sucrose intake (indicator of anhedonia); (ii) tail suspension test (TST); (iii) forced swimming test (FST) (both measuring the degree of failure of escape-like behaviours, indicative of vulnerability to stress); and (iv) open field test (OFT), a measure of exploratory behaviour and anxiety [55–57]. We analysed behavioural tests, considering the number of positive tests (i.e. indicative of depressive-like behaviour) for each animal; for behavioural test thresholds see the Materials and methods section. In the control group, as shown in Fig. 1c, 35% of mice did not show any depressive-like behaviour, 26% of mice showed only one positive test, 26% of mice developed two positive tests, and 13% of mice show three positive tests. None of them showed fully depressive-like behaviours. In the CUMS group, the majority of animals demonstrated depressive-like behaviours, although a sub-population (12%) did not develop such behaviours at all, indicating resistance to stress. In the Ezrin-OE + CUMS mice, in contrast, most of the animals showed resistance to stress, with 25% having all tests negative, 30% showing only one positive test, 30% showing two positive tests, only 15% showing three positive tests and none showing all four tests indicative of depressive-like behaviour. Finally, animals from the Ezrin-KD + CUMS group were more sensitive to stress, with 65% of animals showing three or four positive tests, 20% showing two positive tests, and 15% showing one positive test. None of Ezrin-KD + CUMS animals was fully stress-resistant (Fig. 1c). The sensitivity of mice to CUMS correlated with the expression of Ezrin in the mPFC, quantified by immunocytochemistry (Fig. 1d). Thus we concluded that the level of astrocytic Ezrin defines mouse resilience/sensitivity to chronic stress.

For subsequent astrocyte analysis we selected animals to set up five experimental groups of six mice each. The first (control) was composed from six mice randomly selected from animals not exposed to CUMS. We assigned (again randomly) mice showing four positive tests to the second group (CUMS-sensitive), and (CUMS-resistant) mice, showing no positive tests, were allocated to the third group; the fourth group was made up of Ezrin-OE + CUMS animals showing maximal resistance to stress, and the fifth group included Ezrin-KD + CUMS mice with four positive tests each. We also analysed the behavioural outcome of all mice with Ezrin manipulations (OE and KD) subjected to CUMS. Again, Ezrin overexpression prevented the development of depressive-like behaviours, whereas in Ezrin knockdown mice, behavioural tests were indicative of depressive-like behaviours (Fig. S2).

**Figure 2.**
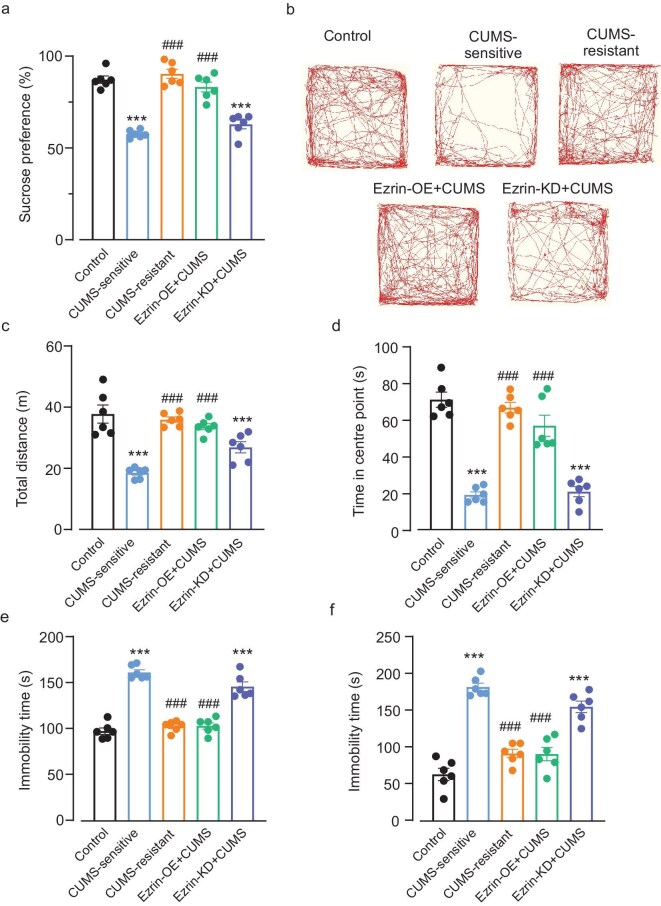
Behavioural phenotypes of mice subjected to the CUMS protocol. (a) Sucrose preference in mice from different experimental groups (one-way ANOVA followed by *post hoc* Tukey test). (b–d) Exploratory behaviour in OFT; (b) representative running trace in OFT, the observation time was 10 min; (c) total running distance (one-way ANOVA followed by *post hoc* Tukey test); (d) centre-point cumulative duration (Kruskal–Wallis test followed by *post hoc* multiple comparison test). (e and f) Immobility time of mice in tail suspension test (one-way ANOVA followed by *post hoc* Tukey test) and FST (one-way ANOVA followed by *post hoc* Tukey test), respectively. All data are presented as mean ± SEM. * versus control group, # versus CUMS-sensitive group. ***/### *P* < 0.001.

The average data obtained from behavioural tests for mouse groups set up as described above are shown in Fig. 2. The sucrose consumption (defined from consumed sucrose solution as a percentage of the total amount of liquid taken in during 12 h that was measured by liquid taken volume), reflecting development of anhedonia, was 87% ± 2% in the control group, 58% ± 0.8% in CUMS-sensitive mice, (*P* < 0.001 vs. control), 90% ± 2% in CUMS-resistant mice (*P* < 0.0001 vs. CUMS-sensitive), 83% ± 3% in the Ezrin-OE + CUMS group (*P* < 0.001 vs. CUMS-sensitive), and 62% ± 2% for the Ezrin-KD + CUMS group (*P* < 0.0001 vs. control) (Fig. 2a).

In the OFT (Fig. 2b and c), the running distance for control mice was 37.74 ± 2.94 m; in CUMS-sensitive mice it was significantly shorter, at 21.30 ± 1.01 m (*P* < 0.0001). In the CUMS-resistant group, the running distance was 35.89 ± 0.89 m, thus being similar to control animals (*P* = 0.9346); likewise it did not differ significantly from the Ezrin-OE + CUMS group (33.59 ± 1.00 m, *P* = 0.4237). In the Ezrin-KD + CUMS group, the running distance was 26.16 ± 1.56 m (significantly smaller than in the control group, and the CUMS-resistant and Ezrin-OE + CUMS groups (*P* = 0.005, 0.0032 and 0.0327, respectively, and not different from CUMS-sensitive mice; *P* = 0.2743). Centre-point cumulative time was 70.78 ± 4.119 s in control mice, 18.89 ± 1.680 s in CUMS-sensitive mice (*P* < 0.0001 vs. control), 66.41 ± 2.894 s in CUMS-resistant mice, 56.55 ± 5.773 s in Ezrin-OE + CUMS mice, and 20.65 ± 2.724 s in Ezrin-KD + CUMS mice. To exclude possible impairment of locomotive function we measured the speed of mouse movements, which did not differ between any of the groups (Fig. S3). The velocities for each group were: control: 4.98 ± 0.24 cm/s; CUMS: 4.89 ± 0.01 cm/s (*P* = 0.9693 vs. control); Ezrin-OE + CUMS: 5.32 ± 0.18 cm/s (*P* = 0.7013 vs. control); Ezrin-KD + CUMS: 5.03 ± 0.20 cm/s (*P* = 0.9988 vs. control).

Depressive-like behaviours assessed with TST and FST (Fig. 2e and f) showed the following outcomes. In control mice, the TST immobility time was 97.83 ± 3.56 s, in CUMS-sensitive mice it was 161.80 ± 2.97 s (*P* < 0.0001 vs. control), in CUMS-resistant mice it was 103.20 ± 2.37 s, and in Ezrin-OE + CUMS mice it was 103.50 ± 3.44 s (both showing no difference to control (*P* = 0.8345 and 0.8045, respectively, but both were significantly shorter than in the CUMS-sensitive group, *P* < 0.0001). In the Ezrin-KD + CUMS group the immobility time was 146.30 ± 5.18 s. In the FST the immobility time for the control group was 63.67 ± 8.31 s, in the CUMS-sensitive group it was 182.50 ± 5.28 s (*P* < 0.0001 to control), in the CUMS-resistant mice it was 92.00 ± 5.72 s, and in Ezrin-OE + CUMS mice it was 91.33 ± 9.04 s (both showing no significant difference from control, *P* = 0.0803 and 0.0915, respectively, and both being significantly shorter than in the CUMS-sensitive group). In the Ezrin-KD + CUMS group the immobility time was 155.70 ± 7.839 s (*P* < 0.0001 vs. control).

### Stress and manipulation of Ezrin expression affect astrocytic morphology

**Figure 3.**
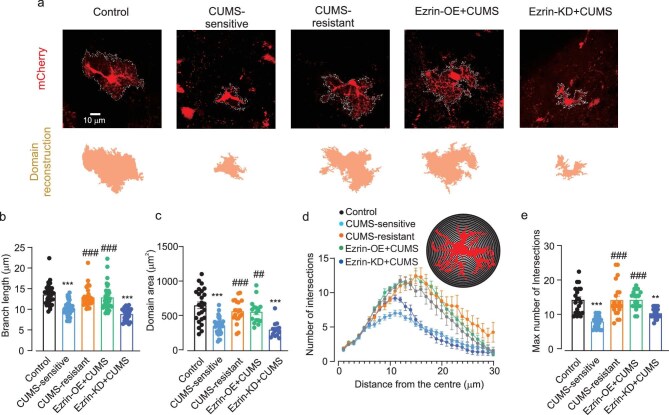
Morphology of mPFC astrocytes from CUMS-treated animals is affected by intrinsic animal sensitivity to stress and by manipulation of astrocyte Ezrin expression. (a) Representative confocal astrocytic images and domain area of astrocytic profiles. (b) Average branch length of astrocyte, *n* = 89–156 cells, 6 mice (Kruskal–Wallis test followed by *post hoc* multiple comparison test). (c) Astrocytic territorial domains, *n* = 93–140 cells, 6 mice (Kruskal–Wallis test followed by *post hoc* multiple comparison test). (d) Sholl analysis of astrocytic morphology shows the number of intersections of astrocytic branches with concentric spheres centred in the middle of cell soma, *n* = 40 cells, 6 mice (two-way ANOVA followed by Dunn’s test). (e) Maximal number of intersections for astrocytes, *n* = 40 cells, 6 mice (Kruskal–Wallis test followed by *post hoc* multiple comparison test). All data are presented as mean ± SEM. * versus control group, # versus CUMS-sensitive group. **/## *P* < 0.01, ***/### *P* < 0.001.

First, we analysed morphology of astrocytes using confocal imaging of astrocytes labelled with cell-targeted genetic indicators (see Materials and methods). Astrocytes from the mPFC of mice sensitive to CUMS showed significant morphological atrophy and loss of complexity (Fig. 3a), evidenced by: (i) a decrease in the length of primary branches (Fig. 3b); (ii) a decrease in astrocytic territorial domains measured as the area of the maximal projection of an astrocyte along the *z*-axis (Fig. 3c); and (iii) a decrease in the number of intersections in the Sholl analysis (Fig. 3d and e). No significant morphological atrophy, however, was detected in mice resistant to CUMS. The size of the territorial domain, the branch length and the number of intersections in control mice were, respectively, 798.4 ± 24.95 μm^2^, 13.94 ± 0.23 μm and 18.00 ± 1.04 in CUMS-sensitive mice these parameters were 347.10 ± 14.79 μm^2^, 10.33 ± 0.20 μm and 10.35 ± 0.42 (*P* < 0.0001 vs. control) and in the CUMS-resistant group they were 675.50 ± 23.77 μm^2^, 13.77 ± 0.28 μm and 15.28 ± 0.56 (*P* = 0.0947, >0.99 and >0.99 vs. control). Ezrin overexpression prevented stress-induced astrocytic atrophy whereas Ezrin-KD + CUMS mice showed astrocytic atrophy similar to that of mice from the CUMS-sensitive group (territorial domain, branch length and number of intersections were 672.50 ± 27.60 μm^2^, 13.33 ± 0.35 μm and 16.63 ± 0.70 in the Ezrin-OE + CUMS group, and 334.10 ± 11.65 μm^2^, 10.21 ± 0.16 μm and 12.58 ± 0.30 in the Ezrin-KD + CUMS group; Fig. 3).

**Figure 4.**
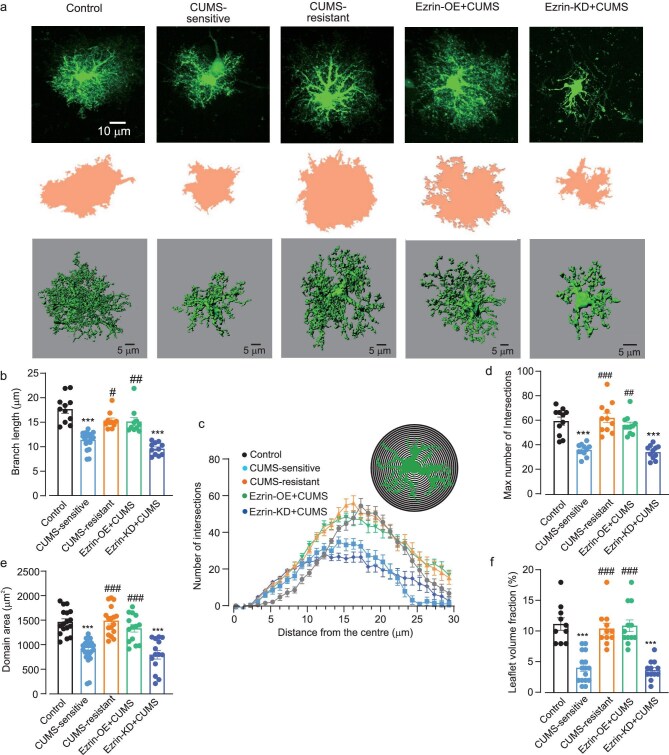
Fine morphology of astrocytes subjected to CUMS. (a) Representative confocal images (top), domain projection (middle) and 3D reconstruction profile (bottom) of Lucifer yellow-labelled astrocytes. (b) Average length of astrocytic processes, *n* = 10–20 cells, 3 mice (Kruskal–Wallis test followed by *post hoc* multiple comparison test). (c) Sholl analysis of astrocytic morphology shows the number of intersections of astrocytic branches with concentric spheres centred in the middle of cell soma, *n* = 11 cells, 3 mice (two-way ANOVA followed by Dunn’s test). (d) Maximal number of intersections for astrocytes, *n* = 11 cells, 3 mice (one-way ANOVA followed by *post hoc* Tukey test). (e) Astrocytic territorial domains, *n* = 14–26 cells, 3 mice (Kruskal–Wallis test followed by *post hoc* multiple comparison test). (f) Astrocytic VF, *n* = 10–16 cells, 3 mice (one-way ANOVA followed by *post hoc* Tukey test). All data are presented as mean ± SEM. * versus control group, # versus CUMS-sensitive group. */# *P* < 0.05, **/## *P* < 0.01, ***/### *P* < 0.001.

To analyse astrocytic morphology in more detail we performed confocal imaging on mPFC astrocytes injected with Lucifer yellow in cortical slices (see Zhou *et al.* [44] and Materials and methods section, for the detailed description of the technique). This approach allows much more insight into fine astrocytic morphology, as shown in Fig. 4, which demonstrates representative 2D images, territorial domains and 3D reconstructions of astrocytes from different experimental groups. In addition, the complexity of astrocytes was assessed by Sholl analysis. Confocal imaging of Lucifer yellow-injected astrocytes further corroborated morphological changes induced by stress and manipulation of astrocytic Ezrin expression. Astrocytes from CUMS-sensitive mice showed prominent morphological atrophy (Fig. 4a–d); whereas CUMS-resistant astrocytes did not differ from the control group (the size of territorial domain, branch length and number of intersections in control mice were, respectively, 1470.00 ± 62.72 μm^2^, 17.64 ± 0.84 μm and 60.00 ± 3.27; in CUMS-sensitive mice these parameters were 897.40 ± 47.58 μm^2^, 11.37 ± 0.39 μm and 36.45 ± 1.49 (*P* < 0.0001 vs. control), while in the CUMS-resistant group they were 1495.00 ± 69.73 μm^2^, 15.28 ± 0.54 μm and 62.64 ± 3.83 (*P* > 0.99, >0.99 and 0.9568 vs. control). Exposure to stress did not significantly alter morphology of the astrocytes from Ezrin-OE + CUMS animals, whereas astrocytes from the Ezrin-KD + CUMS group showed significant atrophy (territorial domain, branch length and number of intersections were 1338.00 ± 76.23 μm^2^, 15.09 ± 0.83 μm and 56.73 ± 2.39; in the Ezrin-OE + CUMS group they were 800.30 ± 88.35 μm^2^, 9.55 ± 0.38 μm and 34.73 ± 1.67). We also quantified the volume fraction (VF [58]) of astrocytic leaflets (Fig. 4f) to assess changes associated with stress and manipulation of Ezrin expression. The VF of optically unresolved processes was estimated as a ratio of fluorescence along the astrocytic anatomic domain cross-section to the maximal fluorescence of soma, as described by Medvedev *et al.* and Popov *et al.* [59,60]. Mean VF was significantly reduced in the astrocytes from CUMS-sensitive and Ezrin-KD mice when compared to control (control: 11.20 ± 1.00; CUMS-sensitive: 4.06 ± 0.60; Ezrin-KD + CUMS: 3.67 ± 0.45; *P* < 0.0001 vs. control for both); in CUMS-resistant and Ezrin-OE + CUMS animals, VF did not differ significantly from the controls (CUMS-resistant 10.45 ± 0.89, *P* = 0.9701; Ezrin-OE + CUMS 10.92 ± 0.97, *P* = 0.9992).

### Ezrin and phosphorylated-Ezrin expression in astrocytes resistant and sensitive to stress

**Figure 5.**
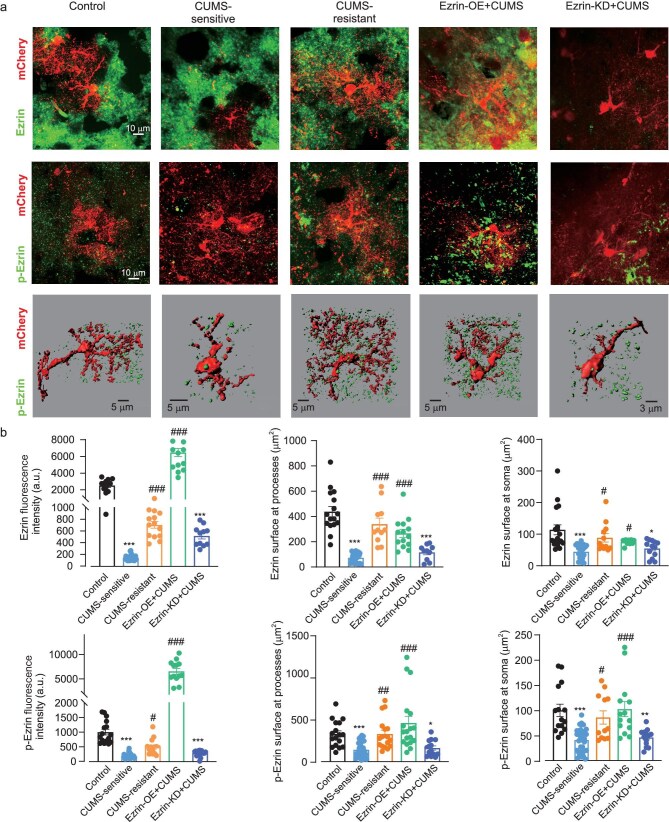
Astrocytic morphology and Ezrin expression: direct correlation. (a) Representative confocal astrocytic images with Ezrin staining (top), p-Ezrin staining (middle); representative 3D-reconstruction (bottom) of astrocytic profiles (red) with p-Ezrin puncta (green). (b) Average fluorescence intensity of Ezrin and p-Ezrin, *n* = 11–24 cells, *n* = 13–32 cells, 6 mice (left column) and surface area of Ezrin and p-Ezrin puncta associated with processes, *n* = 38–67 cells, *n* = 37–68 cells, respectively, 6 mice, (middle column) and soma of 3D reconstructed astrocytes, *n* = 37–79 cells, *n* = 35–40 cells, respectively, 6 mice (right column). All data are presented as mean ± SEM. * versus control group, # versus CUMS-sensitive group. */# *P* < 0.05, **/## *P* < 0.01, ***/### *P* < 0.001. All data were analysed by Kruskal–Wallis test followed by *post hoc* multiple comparison test.

We quantified Ezrin expression with immunoassay against Ezrin and phosphorylated Ezrin (p-Ezrin, the active form of this linker) in all groups. Ezrin was revealed as puncta associated with fluorescently labelled astrocytic profiles (Fig. 5a shows representative images of astrocytes, Ezrin and p-Ezrin immunoreactivity, and 3D reconstructions of astrocytes and p-Ezrin puncta). Exposure to CUMS led to a significant decrease in both Ezrin and p-Ezrin in CUMS-sensitive mice, with no changes in these proteins in CUMS-resistant animals. As expected, Ezrin intensity was high in the Ezrin-OE + CUMS group, and low in the Ezrin-KD + CUMS group. Quantification of expression of Ezrin and p-Ezrin in all experimental groups is shown in Fig. 5b. The colocalization of Ezrin and p-Ezrin with soma and processes of astrocytes quantified as a ratio of total Ezrin surface area versus the total area of astrocytic profile was as follows. Soma: control group 105.30 ± 8.16 μm^2^; CUMS-sensitive group 36.57 ± 3.03 μm^2^ (*P* < 0.0001 vs. control); CUMS-resistant group 98.89 ± 6.88 μm^2^; Ezrin-OE + CUMS group 107.00 ± 8.40 μm^2^ (*P* < 0.0001 vs. CUMS-sensitive); and Ezrin-KD + CUMS 46.23 ± 2.92 μm^2^ (*P* < 0.0001 vs. control). Association of Ezrin with astrocytic arborization showed similar changes and values were: 345.80 ± 23.24 μm^2^ for the control group; 70.70 ± 6.03 μm^2^ for CUMS-sensitive; 293.70 ± 25.72 μm^2^ for CUMS-resistant; 290.80 ± 24.95 μm^2^ for Ezrin-OE + CUMS; and 82.13 ± 6.78 μm^2^ for the Ezrin-KD + CUMS group (Fig. 5b).

p-Ezrin puncta surface to astrocytic soma surface colocalization in the CUMS-resistant group was significantly higher than in CUMS-sensitive mice (CUMS-resistant: 82.97 ± 6.76 μm^2^ vs. 36.93 ± 3.75 μm^2^ in CUMS (*P* < 0.0001); Ezrin-OE + CUMS: 103.40 ± 10.18 μm^2^ vs. 36.93 ± 3.75 μm^2^ in CUMS (*P* < 0.0001); Ezrin-KD + CUMS: 42.76 ± 3.86 μm^2^, (*P* > 0.99 vs. CUMS, Fig. 5b). Colocalization of p-Ezrin with astrocytic branches also showed the same trend when compared with the CUMS-sensitive group; CUMS-resistant: 269.10 ± 23.42 μm^2^ vs. 109.50 ± 14.31 μm^2^ in CUMS (*P* < 0.0001); Ezrin-OE + CUMS: 308.90 ± 39.54 μm^2^ vs. 109.50 ± 14.31 μm^2^ in CUMS (*P* < 0.0001); Ezrin-KD + CUMS: 76.96 ± 8.47 μm^2^ (*P* > 0.99 vs. CUMS) (Fig. 5b).

## DISCUSSION

Astrocytes are a fundamental component of the neural network in the CNS, being indispensable for nervous tissue homeostasis and defence [36,61,62]. In particular, astrocytes regulate neurotransmission and support synaptic connectivity through multiple mechanisms including regulation of synaptogenesis, control of synoptic maturation, support of homeostasis of the synaptic cleft and catabolism of major neurotransmitters, as well as overseeing synaptic elimination [31,34,63,64]. Deficient astrocytic homeostatic support and failed neuroprotection are fundamental pathophysiological mechanisms that contribute to the pathogenesis of numerous neurological and neuropsychiatric diseases [36]. Astrocyte–synaptic interactions are of particular relevance for regulation of neurotransmission in both physiological and pathological contexts. These interactions mainly centre on astrocytic leaflets that establish contacts with synapses, thus contributing to multicomponent synaptic units integrating pre- and post-synaptic neuronal compartments with processes of astrocytes, microglia and oligodendrocyte precursor cells [26,31,65–67]. Morphological plasticity of astrocytic leaflets defines the presence of the latter within the synaptic landscape, and modulates synaptic transmission, thus contributing to the cognitive process [68,69]. Retraction or extension of leaflets significantly affects synaptic kinetics of glutamate, glutamate spillover and K^+^ buffering, thus modulating the course of synaptic transmission and modulating synaptic plasticity [60,70]. In physiological settings astrocytic morphological changes are transient and reversible; in pathology they become rigid and long-lasting thus, arguably, contributing to aberrant synaptic transmission and intimately involved in the pathophysiology of various psychiatric diseases including disorders of mood. Astrocytic morphological atrophy and functional asthenia are fundamental elements of many (if not all) neurological, neuropsychiatric and neurodegenerative disorders [36]. Decreased morphological presence of astrocytes and loss of astrocytic homeostatic function is prominent in normal ageing [38], in cognitive impairments following general anaesthesia [71], in amyotrophic lateral sclerosis [72], in Alzheimer’s disease [41], and in familial migraine [73], to name but a few neurological conditions.

Astrocytic atrophy in depressive disorders and in stress-induced depressive behaviours is well documented [17,53,54]. Causal relationships between the morphological presence and functional activity of astrocytes and behavioural changes are widely recognized, whereas anti-depressant therapies restore astrocytic morphology together with rescuing behavioural deficits [18,21,23]. In the present study we revealed the plasmalemma–cytoskeletal linker Ezrin as a key molecule responsible for astrocytic morphological remodelling in response to chronic stress, and moreover we found that manipulating Ezrin expression in astrocytes from the mPFC affects the susceptibility of mice to stress challenge. We demonstrated that astrocyte-specific Ezrin overexpression markedly increases mouse resistance to stress, and vice versa, down-regulation of astrocytic expression of Ezrin facilitates the development of depressive-like behaviour following exposure to CUMS. In parallel, Ezrin expression correlates with (and arguably defines) astrocytic morphology. Mouse responses to the chronic stress are heterogeneous and not every animal develops depressive-like behaviours; moreover, some mice from the control group not exposed to CUMS showed limited depressive-like behaviours, reflecting a biodiversity continuum. Individual differences in sensitivity to stress have been well established in animal models and reflect human pathology [10,12]. Stress susceptibility and resilience are characteristic features of depressive disorders in patients; the balance between the two is defined by both anatomical and functional idiosyncrasies of every given individual [9,11]. In this respect stress-resilience closely borders the cognitive reserve, which defines cognitive outcome of various neurological diseases [74,75]. The molecular and anatomical background for stress resilience remains largely unexplored. We hypothesized that changes in expression of the plasmalemmal–cytoskeletal linker Ezrin, which belongs to the Ezrin/radixin/moesin family and is expressed predominantly in astrocytic leaflets [30], contributes to stress-induced atrophy of astrocytes.

We characterized in-depth relationships between astrocytic morphology and Ezrin expression in the context of susceptibility to stress. To this end, we segregated the naïve mice into those showing full resistance to stress from those showing maximal sensitivity to it and analysed them separately. We found that neither astrocytic morphology nor Ezrin presence in astrocytes in mice resilient to stress differed substantially from control animals not subjected to stress. Similarly astrocytic morphology was not changed in mice over-expressing Ezrin and subjected to the CUMS protocol. On the contrary, astrocytes demonstrated profound atrophy and decrease in Ezrin expression in CUMS-sensitive naïve animals and in Ezrin-KD mice; all major morphometric parameters such as complexity, size of territorial domain, and branch length were significantly decreased. Astrocytic atrophy was analysed in more detail by imaging astrocytes injected with Lucifer yellow—these images confirmed profound atrophy of astrocytes and revealed a significant decrease in VF occupied by astrocyte leaflets relevant for synaptic coverage. This atrophy was evident in CUMS-sensitive and Ezrin-KD animals; neither CUMS-resistant nor Ezrin-OE mice showed signs of atrophy or a decrease in VF of leaflets.

How can astrocytic atrophy and withdrawal of leaflets contribute to the pathophysiology of major depression? The answer probably lies in the substantial remodelling of the synaptic landscape leading to aberrant neurotransmission. At the same time, retraction of leaflets from synapses modifies glutamate clearance and K^+^ buffering, both contributing to changes in neuronal excitability, information processing and memory [45]. In particular, depressive-like behaviours in animals and depression in patients are associated with a failure in glutamate homeostasis [50,76]. In particular, an increased serum glutamate was detected in patients with MDD [77], and moreover the serum glutamate-positive levels correlated with the severity of depression [77]. Aberrant glutamate homeostasis may also translate into loss of spines and dendritic shrinkage in hippocampal neurones, as well as in decreased neurogenesis [78]. Astrocytes are central for regulation of glutamate levels in the interstitium through the glutamate uptake mediated by the excitatory amino acid transporters EAAT1 and EAAT2 [79]. Retraction of astrocytic leaflets, where the majority of glutamate transporters are concentrated [80], from the synapses compromises glutamate clearance, thus increasing glutamate levels and affecting glutamate homeostasis. Furthermore, reduced astrocytic homeostatic support may directly affect neuronal adaptation and alter neuronal ensembles, which in turn are responsible for behavioural changes. Preventing astrocyte atrophy through cell-specific expression of Ezrin increases stress resilience and reduces emergence of depressive-like behaviours. Thus Ezrin acts as a molecular target that translates stress into behavioural outcome through affecting astrocytes, which in turn precipitate changes in neuronal architecture and aberrant mood. How, indeed, does stress act on astrocytes and what is the molecular link? This requires further investigation. Stress most likely acts on the brain through activation of the hypothalamic–pituitary–adrenal (HPA) axis that affects various aspects of the nervous system including metabolism, hormonal control, excitability and ultimately function [81–83]. How are astrocytes implicated and what is the mechanistic link to astrocytic morphology? The latter is under noradrenergic control [84], which may be affected by activation of the HPA axis. Future focused studies are needed to resolve this matter.

## CONCLUSION

In summary, we demonstrated that astrocytic expression of Ezrin is directly linked to astrocytic atrophy and consequently to the susceptibility of animals to stress. Increase in Ezrin expression prevents astrocytic atrophy and favours stress-resilience; on the contrary, decreased expression of Ezrin promotes atrophy and facilitates development of depressive-like behaviours in response to stress. Our study provides experimental evidence supporting an idea that Ezrin boosts astrocytic presence in the brain active milieu, thus protecting it against stress-induced pathological remodelling resulting in mood disorders.

## MATERIALS AND METHODS

### Animals

All experiments were performed on C57BL/6 mice (obtained from Chengdu Dossy Experimental Animal Co., Chengdu, China); the mice were 6 weeks old at the beginning of the experimental protocol, which lasted for 10 weeks (Fig. 1a). All mice were adapted to the standard laboratory conditions (24°C ± 2°C room temperature and 65% ± 5% humidity on 12/12 h light/dark cycles) with drinking water and food available *ad libitum*. No statistical methods were used to pre-determine sample sizes, but our sample sizes are similar to those reported in previous publications [48,54]. The experimental procedures were in accordance with the National Institute of Health Guidelines for the Care and Use of Laboratory Animals and approved by the Animal Ethics Committee of Chengdu University of Traditional Chinese Medicine (protocol code, AM3520, 8 May 2019).

### Experimental groups

We established five experimental animal groups (to which mice were randomly assigned) before behavioural tests: (i) control group; mice were injected with mCherry construct to label astrocytes, and were not exposed to CUMS; (ii) CUMS group—animals were injected with mCherry construct and were exposed to CUMS; (iii) Ezrin-OE + CUMS Ezrin-overexpression group—animals were injected with both mCherry and Ezrin-overexpression virus and exposed to CUMS; (iv) Ezrin-KD + CUMS group, animals were injected with mCherry and Ezrin knockdown virus and exposed to CUMS. In total, we performed behaviour testing in 166 mice: control group (*n* = 23), CUMS group (*n* = 103), Ezrin-OE + CUMS group (*n* = 20), Ezrin-KD + CUMS group (*n* = 20). All experiments were carried in three separate batches; we found no inert-batch variability (Figs S4–S6).

### CUMS regimen

Mice were exposed to the random sequence of stressors during each 24-h period for 4 weeks, as previously described [23,85]. These stressors included water and food deprivation (12 h), cage tilt of 45° (12 h), group housing (12 h), swimming in 4°C water (5 min), foot shock (1 mA, 5 min), noise (120 dB for 3 h), tail suspension (5 min), damp bedding (12 h), cage shaking (40/min for 5 min) and restraint (1 h). To minimize the effects of stressors on behavioural tests we skipped tail suspension and force swimming in the last week of the CUMS procedure.

### Behavioural tests

#### Sucrose preference test (SPT)

The SPT is a reward-based test and a measure of anhedonia, and it was performed as previously described [86]. The mice were singly caged for 3 days and given two 50 mL bottles containing water or water-based 1% sucrose solution (wt/vol), respectively. The bottle positions were switched daily to avoid a side bias. Following a 24 h period of water and food deprivation, the preference for sucrose or water was determined overnight. Sucrose preference (%) was quantified as [volume sucrose/(volume sucrose + volume water)] × 100%.

#### Tail suspension test (TST)

The TST is a behavioural despair-based test assessing the duration of immobility of mice subjected to inexorable conditions, as previously described [87]. Each mouse was suspended by its tail at a height of 20–25 cm by using a piece of adhesive tape wrapped around the tail 1 cm from the tip. Behaviour was recorded for 6 min. The immobility time was measured during the final 4 min as nearly all mice attempted to escape in the first 2 min [88–90]. The duration of immobility was calculated by an observer blinded to the treatment groups. The mice were considered to be immobile only when they remained completely motionless; mice that climbed along their tails were not included.

#### Forced swimming test (FST)

The FST was performed as previously described [91], in a clear glass cylinder filled with water (temperature, 23°C–25°C); the cylinder’s dimensions were: height, 30 cm; diameter, 20 cm; water level, 15 cm. Mice were gently placed in the tanks. Following the swimming session, the mice were removed from the water by their tails, gently dried with towels, and kept warm under a lamp in their home cages. They were considered to be immobile whenever they stopped swimming and remained floating passively, still keeping their heads above the surface of the water. The time of immobility was analysed during the last 4 min of the 6-min testing period, which followed 2 min of habituation [92].

#### Open field test (OFT)

The OFT was performed as previously described [23]. The apparatus consisted of a rectangular chamber (40 × 40 × 40 cm) made of white, high-density, non-porous plastic. Mice were gently placed in the centre of the chamber and their motility was recorded for 10 min. The total running distance, and the time spent in the centre versus the periphery of the open field chamber were recorded by a camera connected to a computer using an automated video tracking program (EthoVision XT 9.0; Noldus, Wageningen, The Netherlands). The chamber was thoroughly cleaned with 95% ethanol and double distilled water, and dried prior to use and before subsequent tests, to remove any scent clues left by the previous subject.

### Behavioural test thresholds

We set the thresholds for depressive like behaviours based on previously published data [23,48,93]. TST: immobility time of mice of >120 s within 4 min was considered as depressive-like behaviour. FST: immobility time of mice of >110 s within 4 min was considered as depressive-like behaviour. SPT: sucrose preference of <75% was considered as depressive-like behaviour. OFT: total distance of <35 m and centre-point cumulative time of <30 s was considered as depressive-like behaviour.

### Adeno-associated virus (AAV) microinjections

Viral injections were performed 3 weeks before CUMS treatment, as indicated in Fig. 1, by using stereotaxic apparatus (RWD, Shenzhen, China) to guide the placement of a Hamilton syringe fixed with bevelled glass pipettes (Sutter Instrument, 1.0-mm outer diameter) into the mPFC [94]. Animals were anaesthetized with isoflurane (4% for anaesthesia induction, 1% for general anaesthesia). The injection site was located at half of the distance along a line defined between each eye and the lambda intersection of the skull. The needle was held perpendicular to the skull surface during insertion to a depth of approximately 0.2 mm. A total of 0.7 μL of AAV2/8-gfaABC1D-Ezrin-HAx2-P2A-mCherry-WPRE-pA, AAV5-gfaABC1D-mCherry-WPRE-pA [1 × 10^12^ genome copies (gc)/mL; Taitool Bioscience, Shanghai, China], AAV2/8-gfaABC1D-miRNAi (Ezrin)-BFP-WPRE-SV40, or AAV2/8-gfaABC1D-miRNAi(NC)-BFP-WPRE-bGHpolyA (1 × 10^12^ gc/mL; VectorBuilder, Guangzhou, China) was slowly injected into the right side of the mPFC. Glass pipettes were left in place for at least 5 min. After injection, animals were allowed to completely recover under a warming blanket and then returned to the home cage.

### Immunohistochemistry

Mice were perfused with cold paraformaldehyde (PFA, 4% w/v in phosphate buffered saline (PBS) under deep isoflurane (2%, 5 min) and pentobarbitone (1%, 50 mg/kg) anaesthesia. Brains were collected, postfixed and cryoprotected in 30% (w/v) sucrose solution. Brains were cut using a cryostat in 45-μm thick sections; slices were immediately transferred into storing solution (30% w/v sucrose and 30% ethylene glycol in PBS) and kept at 80°C until use. Free-floating sections were incubated for 1 h in saturation solution (6% fetal calf serum in PBS). The sections were then incubated overnight in the same solution complemented with the primary antibody (rabbit anti-Ezrin 1:100; rabbit anti-p-Ezrin 1:100; Cell Signalling, Danvers, MA, USA). After washing in PBS-Tween (PBST) three times, slices were incubated for 1 h at 37°C in saturation solution containing the relevant secondary antibody (goat anti-rabbit Alexa 488; Invitrogen, Carlsbad, CA, USA). After washing in PBST three times, the coverslips were mounted on slides using anti-fade solution (Solarbio, Beijing, China). Confocal microscopy (Olympus, Tokyo, Japan) or normal fluorescence microscopy (Leica, Wetzlar, Germany) were used to obtain images.

### Lucifer yellow injection

Intracellular dye filling was performed on the lightly fixed brain slices from the mPFC of mice using the protocols as previously reported [95]. In the experiments, mCherry-positive astrocytes in the formalin-fixed brain slice that contained mPFC were visualized. 1.5% Lucifer yellow (Lucifer yellow, Merk, Darmstadt, Germany, #67769-47-5) was iontophoresed into astrocytes. After injection, time-lapse imaging (0.1 Hz) was conducted using a confocal microscope (Nikon A1R+, Tokyo, Japan) with a 40× water-immersion objective lens (numerical aperture 0.8) for 5 min. Finally, the brain slices were mounted on a glass slide by using the anti-fade mounting medium and the confocal imaging stacks were collected with a Z-step size of 0.25 μm under confocal microscopy (Olympus, Tokyo, Japan).

### Sholl analysis

Sholl analysis is a commonly used method to quantify astrocyte process complexity [53,96]. All processing steps were performed using image analysis software ImageJ [https://imagej.net/software/fiji/Sholl_Analysis]. In brief, Z-stacks corresponding to the emission spectrum (565–610 nm) of mCherry-labelling; resolution was 512 × 512 pixels (0.25 μm/pixel) on the *x*- and *y*-axes with a step on the *z*-axis (1 μm/frame) were resampled to the same lateral resolution of 0.25 μm/pixel.

### 3D reconstructions

The confocal imaging stacks were collected with a Z-step size of 0.25 μm under a confocal microscope (Olympus, Tokyo, Japan). 3D reconstructions were processed offline using Imaris 7.4.2 (Bitplane, South Windsor, CT, USA), as reported previously [44,97]. The surface–surface colocalization was calculated by an Imaris plugin [44].

### Statistics

All statistical analyses were performed by GraphPad Prism 8. All data were expressed as mean ± SEM of *n* observations, where *n* is the number of animals in behavioural tests, or astrocyte cells from at least three animals. All analysis was single-blind. Data with more than two groups were tested for significance using the one-way ANOVA test followed by the Holm–Sidak test. Multiple comparisons between the data were performed in case of their non-normal distribution, using the Kruskal–Wallis ANOVA on ranks, followed by Tukey’s test. A two-way ANOVA followed by Dunn’s test was performed to compare data obtained in Fig. 3d and Fig. 4c. Significance was defined as *P* < 0.05.

## Supplementary Material

nwaf480_Supplemental_Files
