# Supplementary material for: Astrocyte Ezrin defines resilience to stress-induced depressive behaviours in mice
Source: Natl Sci Rev. 2025 Nov 4;13(2):nwaf480. doi: 10.1093/nsr/nwaf480 (PMC12839543; doi:10.1093/nsr/nwaf480)
Supplement: nwaf480_Supplemental_Files [file nwaf480_supplemental_files.zip › Supplementary Figures-NSR_MS-2024-1562-R3.doc]

Supplementary Figure 1.

Fluorescent images of Ezrin (immunostaining; green) and astrocyte mCherry reporter (fluorescence, red) in the coronal brain slices of mice with astrocyte-specific over-expression or knock-down of ezrin.

Supplementary Figure 2

Behavioral tests of mice from Control, Ezrin-OE_CUMS and Ezrin-KD+CUMS groups.

Sucrose preference test (SPT) in mice from different experimental groups (Kruskal-Wallis test followed by post hoc multiple comparison test). Immobility time of mice in forced swimming test, FST (one-way ANOVA followed by post hoc Tukey test) and tail suspension test, TST (Kruskal-Wallis test followed by post hoc multiple comparison test) respectively). Exploratory behaviour in open field test (OFT) showing total running distance (Kruskal-Wallis test followed by post hoc multiple comparison test), and centre-point cumulative duration (Kruskal-Wallis test followed by post hoc multiple comparison test). All data are presented as mean ± sem. * p < 0.05, ** p < 0.01. ***p < 0.001.

Supplementary Figure 3.

Average movement velocity recorded in open filed test in mice from different experimental groups.

Supplementary Figure 4. Behavioural phenotypes of mice subjected to CUMS protocol in the first batch.

A. sucrose intake in mice from different experimental groups (Kruskal-Wallis test followed by post hoc multiple comparison test). B-C. Immobility time of mice in tail suspension test (Kruskal-Wallis test followed by post hoc multiple comparison test) and forced swimming test (Kruskal-Wallis test followed by post hoc multiple comparison test) respectively). D-E exploratory behaviour in open field test; D. Total running distance (one-way ANOVA followed by post hoc Tukey test). E. Centre-point cumulative duration (one-way ANOVA followed by post hoc Tukey test). All data are presented as mean ± sem. *versus control group, # versus CUMS-sensitive group. */#P< 0.05, **/## P< 0.01,***/###P< 0.001.

Supplementary Figure 5. Behavioural phenotypes of mice subjected to CUMS protocol in the second batch.

A. sucrose intake in mice from different experimental groups (Kruskal-Wallis test followed by post hoc multiple comparison test). B-C. Immobility time of mice in tail suspension test (one-way ANOVA followed by post hoc Tukey test) and forced swimming test (one-way ANOVA followed by post hoc Tukey test) respectively). D-E exploratory behaviour in open field test; D. Total running distance (Kruskal-Wallis test followed by post hoc multiple comparison test). E. Centre-point cumulative duration (one-way ANOVA followed by post hoc Tukey test). All data are presented as mean ± sem. *versus control group, # versus CUMS-sensitive group. */#P< 0.05, **/##P< 0.01,***/###P< 0.001.

Supplementary Figure 6. Behavioural phenotypes of mice subjected to CUMS protocol in the third batch.

A. sucrose intake in mice from different experimental groups (Kruskal-Wallis test followed by post hoc multiple comparison test). B-C. Immobility time of mice in tail suspension test (Kruskal-Wallis test followed by post hoc multiple comparison test) and forced swimming test (one-way ANOVA followed by post hoc Tukey test) respectively). D-E exploratory behaviour in open field test; D. Total running distance (Kruskal-Wallis test followed by post hoc multiple comparison test). E. Centre-point cumulative duration (one-way ANOVA followed by post hoc Tukey test). All data are presented as mean ± sem. *versus control group, # versus CUMS-sensitive group. */#P< 0.05, **/##P< 0.01,***/###P< 0.001.
